# Exploring Psychophysiological Restoration and Individual Preference in the Different Environments Based on Virtual Reality

**DOI:** 10.3390/ijerph16173102

**Published:** 2019-08-26

**Authors:** Tian Gao, Tian Zhang, Ling Zhu, Yanan Gao, Ling Qiu

**Affiliations:** College of Landscape Architecture and Arts, Northwest A&F University, Xianyang 712100, China

**Keywords:** urban green space, preference, human health, virtual reality, EEG, profile of mood state, attention restoration

## Abstract

Accumulated evidence claims that urban green spaces (UGS) have a positive impact on the physical and mental health of humans. However, little information is available to clearly reveal what the most important driving factors are for human psychophysiological restoration. In order to unveil this uncertainty, this study employed virtual reality (VR) technology to investigate the physiological (electroencephalogram, EEG), and psychological (attention, positive mood, negative mood) responses and individual preferences for different urban environments. Participants (120) were recruited and randomly assigned to experience six different types of environments varying in land use and vegetation structures, which were: Grey space, blue space, open green space, partly open green space, partly closed green space, and closed green space. The results showed that the experience of the six environmental types through VR devices had positive restorative effects on the individuals’ attentional fatigue and negative mood; however, all the participants obtained the highest levels of physiological stress restoration when asked to close their eyes for relaxation. The physiological measurements of the EEG showed no significant differences among the selected types of environments. Meanwhile, the results of the psychological measures suggested that only negative mood showed significant differences of change among the six types of environments, and while the partly open green space had the most positive effect on negative mood, the closed green space had the worst. The blue space and partly closed green space received higher recreational preference ratings than the other four environments, while the closed green space received the lowest recreational preference rating. Moreover, the findings showed that there was a strong positive correlation between people’s preferences and the improvement of their positive mood. This indicated that as the popularity of a natural environment increased, so did the benefits of human health and well-being. In addition, this study shows that VR technology may be utilized as a possible surrogate measure to real scenes in evaluating human physiological and psychological restoration in the future. The present findings can provide the theoretical basis and practical guidance for future optimal planning of urban restorative environments.

## 1. Introduction

As urbanization continues at an alarming rate, it not only threatens biodiversity but also increasingly separates people from the natural environment [1], which is directly linked to undesirable social-related health diseases such as obesity, diabetes, depression, and mental fatigue [2,3]. During the last decade, many studies have demonstrated a positive association between nature and individuals’ health [4,5,6]. Urban green spaces (UGS), which are mainly composed of natural environments, are an important barrier between healthy and unhealthy lifestyles [7]. Hence, the notion of creating UGS that optimize their restorative effects has become a key focus for UGS planning in densely populated areas [8,9].

To date, several studies have shown that UGS have restorative effects for inhabitants, such as attention recovery [10,11], stress, and mental fatigue restoration, mood promotion, and the prevention of depression [10,12,13,14]. Two broadly accepted theoretical frameworks that explain how green environments affect human health and well-being are the attention restoration theory [11] and stress reduction theory [15]. Despite the wide applications of these two theories in UGS studies, effective guidelines for designing a restorative environment are lacking [2]. This is because the dominant characteristics of restorative environments are unclear, especially for UGS [16]. Therefore, it is necessary to identify which structural components of the UGS are the most important driving factors for human health [16,17]. To address this, the present study examined the restorative effects of UGS differing in characteristics, based on vegetation structures [18,19,20].

It is worth noting that whilst it is generally realized that UGS have restorative effects, there is evidence that preferences have played an important role in attracting people to restorative environments and in keeping them in such environments for a prolonged period of time; that is, individual preference may be a keystone for the restorative action of environments. Several studies have identified a positive relationship between aesthetic preference and mental restoration [9,21,22,23,24]. However, not all specific types of UGS facilitate individual preference [25]. Hoyle et al. and Han demonstrated that an environment with high restoration is not always a high aesthetic quality environment that garners high preference [16,26]. Given these inconsistent results, the relationships between recreational preferences and restoration in the different environments require further examination.

Psychophysiological responses can often be elicited by the visual perception of environmental stimuli [27]. Most empirical studies have employed subjective rating scales to examine the environmental effects on human health, while objective measurements have rarely been used [28,29]. The electroencephalogram (EEG) can be used to detect an individual’s electrical activities in the brain, and is considered an objective indicator of the physiological stress response in humans [30,31]. The spontaneous bioelectrical signal of the brain can be divided into alpha waves (8–13 Hz), beta waves (>13 Hz), delta waves (equal to 4 Hz), theta waves (4–8 Hz), and other types according to the different frequencies of the EEG [30]. Hankins et al. found that when stress or fatigue increased, the EEG alpha waves vanished and changed into beta waves and theta waves, which indicated that the EEG alpha waves were prominent in a state of relaxed wakefulness [32,33,34]. Ulrich also showed that alpha waves resulting from the exposure to natural photos were significantly higher than those of exposure to townscape photos [35]. Chang et al. found that the EEG alpha waves of a simulated landscape with a restorative function were increased compared to other simulated landscapes [36]. The previous studies showed that the increase of these EEG alpha waves’ value can reflect the physiological relaxations experienced when one is exposed to natural features. Therefore, this study used the EEG alpha waves as a primary indicator of physical relaxation in the experiments.

In addition, compared with the traditional on-site survey that is often influenced by uncontrollable factors such as traffic noise, graffiti, litter, etc. [37], virtual reality technology (VR) is a new way to maximize the perception of an environment by isolating the visual and auditory sense of the outside world, using a head-mounted display device [38]. This guides the user in creating a sense of immersion in a three-dimensional environment, and gives them a better sense of the environment [39]. Accordingly, this study used VR technology as visual stimulation, integrating it with ecological and social methods to explore the effects of the different types of environment on human psychological and physiological health. The specific objectives were to investigate:

1. What is the difference in the restorative state before and after visual stimulation by using VR devices?
2. What are the effects of the different types of environments on people’s physiological and psychological restoration?
3. Which types of environments do people prefer? How do people’s preferences relate to restorative effects of environments?

## 2. Materials and Methods

### 2.1. Stimulus of Experimental Images

**Figure 1:**
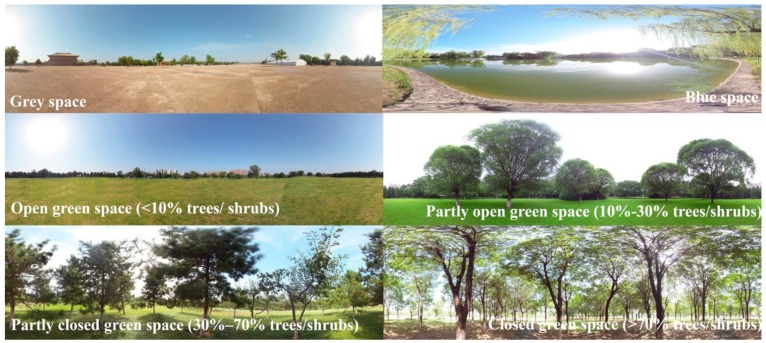
Example panoramic sample photographs of each type.

The visual stimulus materials were VR panoramic photographs, which were shot by a panoramic camera (Insta360 Pro-I) in different urban environments of China. The resolution of the panoramic photographs is 7680 × 3840 (8K) pixels. Visual stimuli were displayed by VR glasses (Pico Goblin VR all-in-one) with a resolution of 2560 × 1440 pixels, and a screen refresh rate of 70 Hz (<20 ms). As shown in Table 1, the selected visual stimuli were classified into the three types of environment according to land use, including grey space, blue space, and green space. UGS were further divided into four types based on the horizontal vegetation structures according to their vegetation patterns and plant configurations, which were open green space, partly open green space, partly closed green space, and closed green space (Table 1). A sorting task procedure was adopted to ensure the selected experimental stimuli were matched according to a specific type of environment. Ten landscape architecture experts were invited to evaluate and sort the 240 panoramic photographs (40 photographs for each type) into six types of environment according to the classification system, and then to rank each photograph within a type based on the size, shape, location, and composition of vegetation structure (Table 1). The evaluation resulted in the selection of the top five panoramic photographs with high scores for each type of environment, amounting to 30 photos (Figure 1).

### 2.2. Participants

In this study, 120 Chinese college students were voluntarily recruited through on-site invitation and online invitation by WeChat (mean age = 20.7, SD = 2.13, 58 males and 62 females). They were healthy with myopia degrees of less than 800 degrees, which was the maximum required degree of the VR glasses. Participants were assumed to have accumulated fatigue through two hours of classroom learning immediately prior to participation. Groups of 20 participants were randomly assigned to one of the specific types of environment for visual evaluation.

### 2.3. Measurements

#### 2.3.1. Physiological Stress

An EEG can be used to measure the brain’s response to external visual stimuli. This study used the NeuroSky portable brainwave device with a NeuroSky TGAM brain wave chip inside to measure human physiological responses through electrodes attached to the scalp; to obtain the EEG data of the participants, the brain wave data were sent to a computer in real time. The EEG alpha waves (8–13 Hz range) obtained can be used as a primary indicator for physiological stress [40], as higher values of the EEG alpha waves indicate better restoration of physiological stress [41].

#### 2.3.2. Psychological Stress

The study measured participants’ stress through the 40-item Profile of Mood States (POMS-SF) scale, which has been most widely used in restorative research, and as a tool for evaluating the psychological stress of an individual [42]. Participants were asked to respond on a 5-point Likert-type scale (1 = not at all; 5 = feel very strongly) of POMS-SF before and after visual stimulation. The scale reveals seven affective measures including tension, anger, fatigue, depression, vigor, esteem, and confusion, which can be classified into two broad emotional dimensions: “Negative mood” (calculated as weighted averages of anger, tension, fatigue, confusion, and depression subscales, and the minimum possible score is 6.08, the maximum possible score is 30.42) and “positive mood” (calculated as weighted average of vigor and esteem scales, and the minimum possible score is 5, the maximum possible score is 25). A higher score for each dimension corresponds to a higher emotional level.

#### 2.3.3. Attention

The Stroop color task was used to assess the restoration of participants’ attention before and after visual stimulation. The Stroop color task is an effective and reliable selective attention measure [43], which is often used for restorative research experiments. In this experiment, the Stroop color task was used for measuring the attention capacity of participants within 45 s, in accordance with the result of the preliminary experiment. Twenty participants were recruited to conduct the Stroop color task first, in order to ascertain an appropriate length of time for the new participants to fulfill the Stroop task successfully in the formal experiment. They were presented with 70 color words printed in incongruent colors, and asked to name the ink color and to complete the task within a specified time [43]. For example, when the word “yellow” is presented in red ink, the participant must say “red”. Only the responses that were correctly completed in 45 s were recorded. The attentional capacity of the participants was calculated by a percentage based on the correct number of responses out of 70 items. The higher the percentage received, the better the attention capacity of the participant.

#### 2.3.4. Preference

The participants’ preferences for each panoramic photo of the environment and their reasonings were collected through a questionnaire survey. For each panoramic photo of one specific type of the environment, the respondents were asked to indicate whether they preferred the environment while wearing VR glasses, using a “Yes/No/Hard to say” response. The latter response was added to provide some indication of how well the environment was perceived by the respondents through VR glasses, as a high percentage of “Hard to say” answers was likely to indicate that the environment demonstrated by VR glasses was less well accepted. In the subsequent statistical analysis, the “Hard to say” answers were treated as “No” in accordance with Wang, and the score assignment was thus “No/Hard to say” = 1 and “Yes” = 2, correspondingly [44].

### 2.4. Design and Procedure

In order to eliminate the nervousness of the participants and to better allow them to understand the experimental process, the procedure, and the relevant equipment’s function within the experiment, were introduced for participants before the experiment began. Each set of experiments in this study was conducted in the afternoon or evening after the participants endured at least 2 h of class time, which induced an accumulation of fatigue in the participants. Each group of participants was asked to see only one type of the panoramic photos at a time.

**Figure 2:**
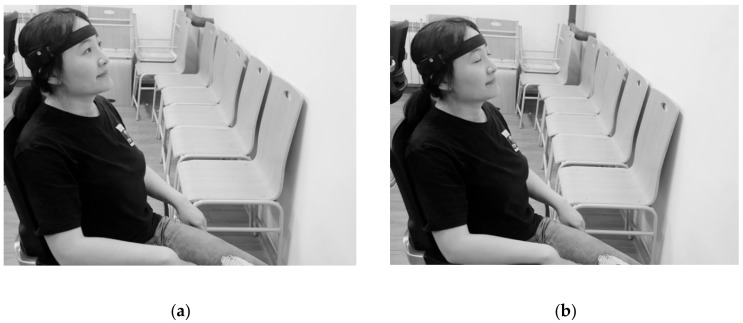

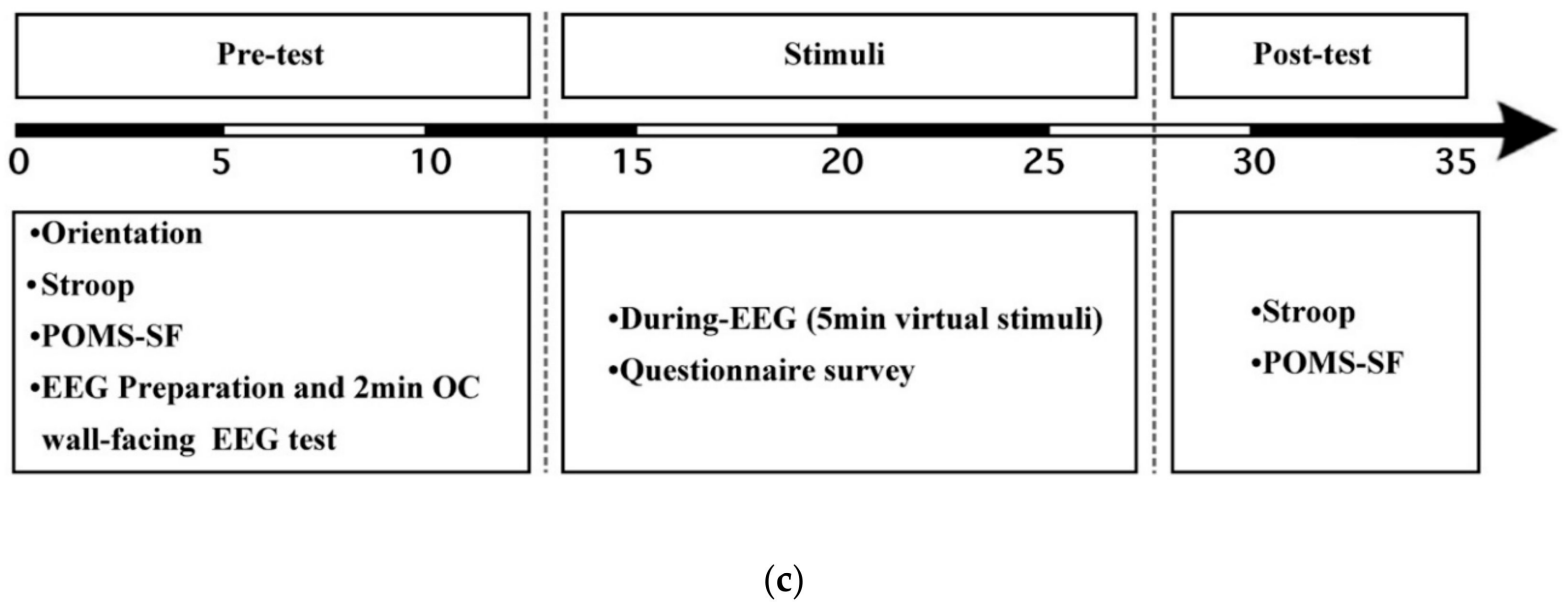
(**a**) One-minute open eye state of electroencephalogram (EEG) data collection; (**b**) one minute closed eye state of EEG data collection; (**c**) study procedure.

First, participants were asked to complete the tests for baseline measures of attention and psychological stress, by using the Stroop color task within 45 s, and then completing a POMS-SF questionnaire. The portable EEG electrode was placed onto participants’ foreheads, and they were asked to sit facing the wall to temporarily exclude external visual stimuli. Then, they were asked to open and close their eyes alternately in two one-minute cycles to determine their brainwave baseline, in order to identify the baseline of psychological stress (Figure 2a,b).

Next, the VR glasses were used to play panoramic photo slides for each participant, corresponding to his/her group assigned. To ensure that the testing time was not too long, the participants could fully experience the scene in the VR glasses. Each slide show contained 5 photos and each photo was displayed for 1 min, with the entire watching time lasting 5 min. All participants’ EEG data were collected during the watching time. Then, the VR glasses and portable EEG electrode were removed from participants. The preference questionnaire for each panoramic photo was then filled out.

Finally, a second round of measuring of attention and stress was completed, consisting of the Stroop color task and the POMS-SF questionnaire. It required approximately 35 min for the whole experimental procedure (Figure 2c).

### 2.5. Data Analysis

The final number of participants was reduced to 116 due to missing brainwave data. SPSS 25.0 software (IBM, Armonk, NY, USA) was used in all statistical analyses. In order to detect differences in the restoration of people’s attentional fatigue and psychophysiological stress before and after viewing the natural environment through VR devices, one-way analysis of variance (ANOVA) was used. To determine how different types of environment affect people’s attention and mood, an analysis of covariance was used, in which pre-tests were regarded as covariates to compare with post-tests of attention and mood in groups exposed to different types of environment. ANOVA was also used to compare the effects of the different types of environment on participants’ physiological stress and recreational preferences. Correlation analysis was employed to explore the relationship between the participants’ recreational preferences and the restoration factor of the environments. The preference was determined by the average score of 116 respondents for the environment they perceived. The restoration of the environment was divided into three variables: ‘attentional fatigue’, ‘physiological stress’, and ‘psychological stress’. Psychological stress was subdivided into negative and positive mood. Each variable was judged by the average score of relevant tests of the participants.

## 3. Results

### 3.1. Restorative State from ANOVA

**Figure 3:**
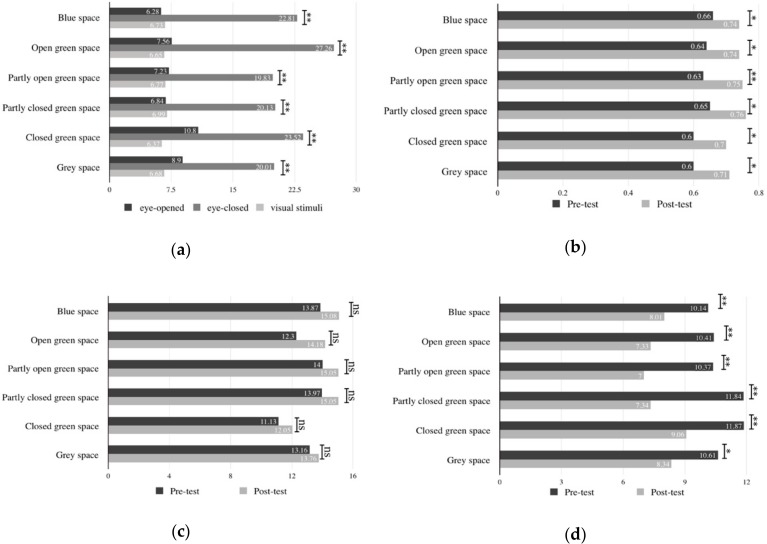
The mean values of psychophysiological indicators before and after the visual stimulation by virtual reality (VR) glasses. Note. ** *F* is significant at the 0.01 level. * *F* is significant at the 0.05 level. ((**a**): EEG alpha, (**b**): attention, (**c**): positive mood, (**d**): negative mood).

The results showed that using the VR glasses as visual stimulation could significantly restore the attentional fatigue and negative mood of the participants, but it did not affect positive mood significantly (Figure 3b–d). The visual stimulation period of the EEG alpha waves was significantly lower than the state when the eyes were closed, but did not significantly differ from the open-eye state (Figure 3a), which means that lower stress was measured when participants’ eyes were closed.

In general, although using VR glasses for visual stimulation could bring a better restorative experience on attentional fatigue and negative mood, the result showed that respondents could obtain better restoration of physiological stress when they closed their eyes to rest.

### 3.2. The Restorative Effects of the Different Environments on Psychophysiological Stress and Attentional Fatigue

There were no significantly different impacts on the restoration of positive mood (*F* = 0.99, *p* = 0.42), attentional fatigue (*F* = 0.39, *p* = 0.85), and physiological stress (*F* = 0.38, *p* = 0. 86) among the six types of the selected environments. Conversely, the post hoc results showed that the six types of environments had significant differences in the restorations of negative mood (*F* = 2.57, *p* = 0.03) (Table 2). Partly open green space (POG) had the most effective reduction of negative mood, followed by open green space (OG), partly closed green space (PCG), blue space (BS), and grey space (GrS), while closed green space (CG) had the least.

### 3.3. Individual Preference and Keyword Analysis of Motivation on Preference in the Different Environments

**Figure 4:**
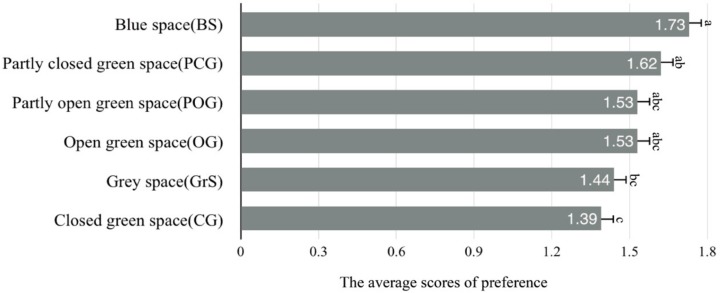
ANOVA analyses of the comparing preferences across the experimental groups.

Preferences for the different types of environment were significantly different (*F* = 5.87, *p* < 0.01), as BS received the highest scores for preference, followed by PCG, POG, and OG. GrS and CG were the least preferred (Figure 4).

### 3.4. Correlations between Recreational Preference and Restorative Effects

**Figure 5:**
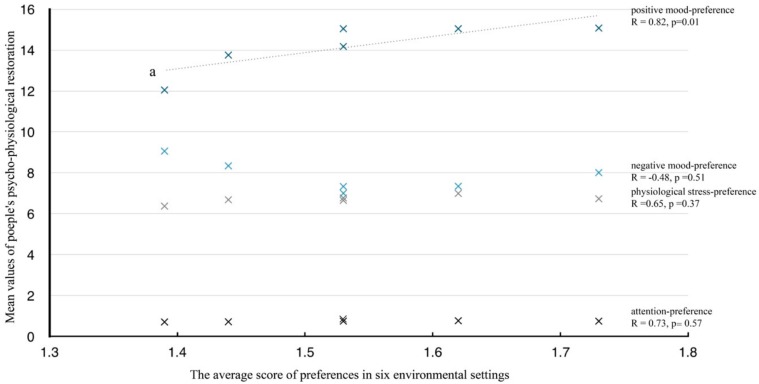
The relationship between preference and restorative effects. Note: “a” represents the trend line of relationship between positive mood and preferences.

The correlation analysis showed that there was a significant correlation between recreational preference and positive mood (*R* = 0.82, *p* < 0.05; Figure 5), which means that the participants’ positive moods can be induced when perceiving the environment they like. However, there were no significant correlations between preferences and the restoration of attention (*R* = 0.73, *p* > 0.05), negative mood (*R* = −0.48, *p* > 0.05), and physiological stress recovery (*R* = 0.65, *p* > 0.05).

## 4. Discussion

### 4.1. What Is the Difference in the Restorative State before and after Visual Stimulation by Using VR Devices?

This study compared the restorative states of participants’ psychophysiological stress and attentional fatigue before and after viewing the panoramic photos of the six types of environment with VR devices. It was found that both negative mood and attentional fatigue had significant restorations after the visual stimulation across all types of the selected environments. This means that participants gained some restorative effects against attentional fatigue and negative mood [5,6], not only from exposure to the natural-like environments (e.g., green and blue space) but also against expectations, from exposure to grey spaces. However, it should be noted that the selected grey space was a public square, which is totally different from traditional urban built environments, in that it is significantly used for community gatherings in spite of its hardscape design, with impervious pavements and a landscape with a mosaic of lawn, sporadic trees, and manicured shrubs. The findings indicate that contact with all six environment types through VR exposure might provide some restoration of attentional fatigue, and some alleviation of negative mood.

However, this study showed that positive mood might not be improved significantly by any of the types of the selected environments. The findings align well with those of Tyrväinen et al., who found that there were no significant differences in the stimulation of positive moods, regardless of whether one was exposed to the natural environment or built environment [31]. Perhaps this is because participants were allotted 5 min to experience the visual stimuli of each environment, and thus the time allotted was too short for the respondents to enhance their positive moods [31]. Another explanation is that the panoramic photos of all of the environments represented traditional parks in China, and we can assume that the participants would not be unfamiliar with such environments in their daily life. Therefore, the selected images might not have been sufficiently novel so as to stimulate their brains in order to produce more chemicals such as dopamine and encephalin, to awaken higher positive emotions from the perspective of neurobiology [45].

Still, it was surprising that closing eyes for only one minute increased ratings of EEG alpha waves significantly, when compared to the visual stimulation period and viewing an empty wall. This finding was not in line with previous studies, which showed that people’s physiological indicators would be improved to some extent, compared with those before viewing the landscape [31,46]. The explanation for this inconsistency might be that during the closed-eyes condition, the participants were asked to relax as much as possible without thinking about anything else. Previous studies indicated that the eyes-closed resting condition could reduce cerebral cortical activity by isolating external interference [47]. At this time, people were experiencing a higher state of relaxation, and their brain waves were mainly dominated by EEG alpha waves [48,49]. The second explanation might be merely an indication of the difference in the cognitive load involved in the visual processing of the panoramic photos, versus the absence of this cognitive load. Onishi and Hagawara found that it is only when the eyes are open that people can infer a degree of cognitive workload [50]. Barry et al. [49] indicated that eyes-closed and eyes-open conditions provide EEG measurements of different environments as well as power levels, and that other brain waves are activated separately during the eyes-open conditions. Therefore, further examination and consideration is required in choosing EEG alpha and eyes-closed conditions as physiological indicators and baseline conditions, for the different paradigms as well in future EEG studies.

### 4.2. Do Different Types of Environment Influence People’s Physiological Stress, Attentional Fatigue, and Mood Differently?

The study identified no difference in restorative effects in physiological stress, attention, and positive mood across the six types of environment. This finding supported the other experimental studies, which have also found few differences in restorative effects among the different types of natural settings [31,51]. That is possibly because the current study’s visual stimulations were selected from urban environmental settings with high levels of human management. These visual stimulations could be more familiar to respondents than other particular environmental settings, such as forests with dense vegetation, nature reserves, etc. [48]. Tyrväine et al. claimed that physiological stress and attentional fatigue stress can be alleviated by an environment with certain prominent characteristics [31]. However, this study showed that the six types of environments had significant differences in the restorative effects on negative mood. It might be explained that negative emotions are more sensitive to the change of environment, which may easily and instinctively improve unpleasant feelings [45]. Our results also showed that the most effective environment to improve negative mood was partly open green space (POG). The possible explanation is that POG had more natural elements, which increased the species richness and complexity of the space; yet would not completely block the participants’ sight and make the environment congruent. Berlyne found that exposure to such environments could inspire higher levels of emotional arousal, thereby reducing negative emotions [52]. Another explanation of the increased mood concerns the biological quality of POG, wherein the plants were tall and robust with pretty shapes. These features could provide better aesthetic experiences and positively improve one’s negative mood [27]. Moreover, this study found that blue space was not as successful as the green spaces on the restorative effects on negative emotions, but that it was better than grey space, although some previous studies have shown that blue space was the supreme place for people to experience mental restoration [53,54]. Perhaps the difference in findings is because through VR devices, participants could only see the water, but couldn’t interact with the environment in a physical way, such as by walking around and approaching it, which is likely to influence the effect of restoration.

### 4.3. What Types of Environment do People Prefer and What Aspects Affect Individuals’ Preference?

In the present study, the result showed that the blue space had the highest level of preferences relative to the green space and the grey space. This confirmed the main results of previous studies on landscape preferences, which indicated that the environments with water were always associated with higher preferences and more positive subjective reactions [38,55]. From an evolutionary perspective, hydrophilia is an innate attribute of humans, which makes people naturally attracted to water (e.g., one of the comments from the participants: ‘I like water very much and it makes me feel peaceful’).

As to green space, the results showed that partly closed green space (PCG) and partly open green space (POG) environments were more liked than open green space (OG) and closed green space (CG). This could indicate that participants disliked overly high or low green coverage levels of space compared to the moderate green coverage. The reason could be explained as follows: first, PCG and POG can receive more sunlight for supporting numerous species, and potentially provide people with more visual accessibilities and aesthetic experiences due to complex vegetation structures, with a moderate canopy cover of trees and shrubs [9,48]. Petucco et al. demonstrated that better visual accessibility was an important factor that could influence recreational preference, because it can increase the legibility of the environment and creates feelings of safety for individuals [56]. For example, participants commented ‘there are some tree trunks with no sheltering from sight but can provide the shelter’, and ‘[I] have [a] sense of safety’. Second, according to the prospect refuge theory, people always prefer environments with obstruction elements, which can allow them to see others without being seen. The open space would be less preferred due to a lack of obstacles and refuges. It is surprising that the closed green space (CG) received the lowest preferences—lower even than the grey space did. A possible explanation could be that the CG had a high level of canopy cover with trees and shrubs, which may give rise to feelings of unsafety and result in untidy, ugly, or even frightening feelings of such environments [57]. Some typical comments in this case were: ‘This place made me feel scary’; ‘My view was blocked’; ‘The landscape was not coherent’. Other possible explanations might be due to the low level of understory management [58]. Nassauer argued that people’s preferences are often ascertained by their picturesque notion of nature that looks tended and tidy, rather than wild, because our cultural expectations of nature demand a certain level of visible care and order [59]. An example of comments included ‘too many fallen leaves’; and ‘I felt depressed because of the short height of the trees with dark colors’. Therefore, an environment with a moderate level of vegetation can better provide the appropriate visual stimulation for human recreational preferences [27].

### 4.4. How do People’s Preferences Relate to the Restorative Effects of Environments?

The results showed that preferences and positive moods were significantly correlated, which means that the exposure to a more preferred environment was associated with more positive changes in positive mood state, including ‘self-esteem’ and ‘vigor’. This finding advocated the speculation that a good experience of aesthetic preference associated with nature may improve individuals’ positive moods [45], although negative mood, attention, and physiological stress were not strongly statistically associated, perhaps due to the limited samples. However, it still can be concluded that the potential trend of the relationship between negative mood and preference was negative, while the relationship with concentration and recovery of physiological stress was positive. Similar results have also been found by previous studies, which suggested that individuals’ preferences for landscapes may facilitate restoration from the directed attention fatigue and physical as well as mental stress [9,24]. That is because it is easy for people to accept a place they like where they can have greater experiences of comfort, safety, and being away from daily life [60], which promotes their physical and mental health [21]. Therefore, it is necessary to create more natural environments with moderate human management and maintenance, such as partly open/closed green spaces and blue spaces, in order to enhance the restorative quality of urban environments.

### 4.5. Limitations and Future Research

There are some limitations in this study. First, this study examined the changes in psychophysiological responses to the different types of environments in a short period of time, so the validity of the results needs to be further explored using a prolonged timeframe. Second, although VR technology could be utilized as a surrogate measure to represent authentic scenery, some specific indexes and standards of VR devices (e.g., panoramic photo pixels, location of viewfinder, wearable portability) need to be improved to avoid cybersickness, so as to obtain more accurate information. Third, the present study only dedicated the subdivision of green space. Grey space and blue space can also be further classified according to the canopy cover ratios with trees or shrubs. Fourth, as for measurement of attentional fatigue, the present study only measured the number of Stroop task items recorded within a timeframe, which could obtain less variability, and thus less of an effect, compared with those measured Stroop versions that integrated speed and accuracy [61]. Additionally, all the participants in the study selected were college students, which could limit the generalization of the results. Further investigation using different green space users and different environmental contexts, and with more comprehensive measurements of attentional fatigue should be examined.

## 5. Conclusions

This study employed VR technology to investigate the effects of six different types of environment on participants’ psychophysiological responses as well as participants’ preferences. The results are described as follows: First, there existed restorative effects on attentional fatigue and negative mood after using VR devices to view the different sceneries. Second, partly open green space (POG) had the most significant effect on negative mood regulation. Third, there was a strong positive correlation between preference for an environment and the improvement of positive mood, as well as a certain correlation with other restorative outcomes, but not all types of UGS were equally shown to have better restorative effects. The results lead to the conclusion that in order to enhance the restorative quality of urban environments and to meet the inhabitants’ recreational needs, spatial configuration and the type of environment should be taken into consideration. More constructions of blue spaces and of specific types of green space, such as POG and PCG, should be encouraged in future.

## Figures and Tables

**Table 1:** Classification of urban environment based on land use and vegetation structures.

Level 1	Level 2	Characteristics of Each Environment
Grey Space (GrS)	-	Open public square (90–100% abiotic area with little greenery)
Blue Space (BS)	-	Open pond (90–100% water with some greenery)
Green Space (GS)	Open green space (OG)	Canopy cover dominated by less than 10% trees/shrubs
	Partly open green space (POG)	Canopy cover dominated by 10–30% trees/shrubs
	Partly closed green space (PCG)	Canopy cover dominated by 30–70% trees/shrubs
	Closed green space (CG)	Canopy cover dominated by more than 70% trees/shrubs

**Table 2:** Covariance analysis of the effect of different environment on the participants’ attention and mood during the pre-tests and post-tests (dependent variable: post-tests).

Source	Sum of Squares	*df*	Mean Square	*F*	Sig.	Partial η2	Post-hoc
**Attention**							
Types of scenery	0.02	5	0.00	0.39	0.85	0.02	
Pre-test	0.87	1	0.87	107.93	0.00	0.50	
Error	0.88	109	0.01				
*R*^2^ = 0.512 (Adj *R*^2^ = 0.49)							
**Negative Mood**							
Types of scenery	49.88	5	9.98	2.57	0.03	0.11	CG ^1^ > GrS ^2^, BS ^3^, PCG ^4^, OG ^5^ > POG ^6^
Pre-test	64.06	1	64.06	16.47	0.00	0.13	
Error	424.01	109	3.89				
*R*^2^ = 0.223 (Adj *R*^2^ = 0.18)							
**Positive Mood**							
Types of scenery	76.18	5	15.24	0.99	0.42	0.04	
Pre-test	65.93	1	65.93	4.32	0.04	0.04	
Error	1664.39	109	15.26	0.99	0.42	0.04	
*R*^2^ = 0.085 (Adj *R*^2^ = 0.04)							

^1^ CG: Closed green space; ^2^ GrS: Grey space; ^3^ BS: Blue space; ^4^ PCG: Partly closed green space; ^5^ OG: Open green space; ^6^ POG: Partly open green space.

## References

[1] Colléony A., White R., Shwartz A. (2019). The influence of spending time outside on experience of nature and environmental attitudes. Landsc. Urban Plan..

[2] Van den Berg A.E., Jorgensen A., Wilson E.R. (2014). Evaluating restoration in urban green spaces: Does setting type make a difference?. Landsc. Urban Plan..

[3] Soga M., Gaston K.J. (2016). Extinction of experience: The loss of human-nature interactions. Front. Ecol. Environ..

[4] Nielsen T., Hansen K. (2007). Do green areas affect health? Results from a Danish survey on the use of green areas and health indicators. Health Place.

[5] Douglas I. (2012). Urban ecology and urban ecosystems: Understanding the links to human health and well-being. Curr. Opin. Environ. Sustain..

[6] Wood L., Hooper P., Foster S., Bull F. (2017). Public green spaces and positive mental health—Investigating the relationship between access, quantity and types of parks and mental wellbeing. Health Place.

[7] Gao T., Song R., Zhu L., Qiu L. (2019). What Characteristics of Urban Green Spaces and Recreational Activities Do Self-Reported Stressed Individuals Like? A Case Study of Baoji, China. Int. J. Environ. Res. Public Health.

[8] Stigsdottera U.K., Corazona S.S., Sideniusa U., Refshaugeb A.D., Grahnc P. (2017). Forest design for mental health promotion—Using perceived sensory dimensions to elicit restorative responses. Landsc. Urban Plan..

[9] Wang R., Zhao J., Meitner M.J., Hu Y., Xu X. (2019). Characteristics of urban green spaces in relation to aesthetic preference and stress recovery. Urban For. Urban Green..

[10] Hartig T., Evans G.W., Jamner L.D., Davis D.S., Gärling T. (2003). Tracking restoration in natural and urban field settings. J. Environ. Psychol..

[11] Hartig T., Mang M., Evans G.W. (1991). Restorative effects of natural environment experiences. Environ. Behav..

[12] Kaplan R., Kaplan S. (1989). The Experience of Nature.

[13] Tsunetsugu Y., Lee J., Park B.J., Tyrväinen L., Kagawa T., Miyazaki Y. (2013). Physiological and psychological effects of viewing urban forest landscapes assessed by multiple measurements. Landsc. Urban Plan..

[14] Moritaa E., Fukudaa S., Naganob J., Hamajimac N., Yamamotod H., Iwaie Y., Nakashimaf T., Ohirag H., Shirakawaa T. (2007). Psychological effects of forest environments on healthy adults: Shinrin-yoku [forest-air bathing, walking] as a possible method of stress reduction. Public Health.

[15] Ulrich R.S., Simons R.F., Losito B.D. (1991). Stress recovery during exposure to natural and urban environment. J. Environ. Psychol..

[16] Hoyle H., Hitchmough J., Jorgensen A. (2017). All about the ‘wow factor’? The relationships between aesthetics, restorative effect and perceived biodiversity in designed urban planting. Landsc. Urban Plan..

[17] Gascon M., Triguero-Mas M., Martínez D., Dadvand P., Forns J., Plasència A., Nieuwenhuijsen M. (2015). Mental health benefits of long-term exposure to residential green and blue spaces: A systematic review. Int. J. Environ. Res. Public Health.

[18] Krause G., Bock M., Weiers S. (2004). Mapping Land-Cover and Mangrove Structures with Remote Sensing Techniques: A Contribution to a Synoptic GIS in Support of Coastal Management in North Brazil. Environ. Manag..

[19] Löfvenhaft K., Björn C., Ihsea M. (2002). Biotope patterns in urban areas: A conceptual model integrating biodiversity issues in spatial planning. Landsc. Urban Plan..

[20] Gao T., Hedblom M., Emilsson T., Nielsen A.B. (2014). The role of forest stand structure as biodiversity indicator. For. Ecol. Manag..

[21] Herzog T.R., Maguire C.P., Nebel M.B. (2003). Assessing the restorative components of environments. J. Environ. Psychol..

[22] Nordh H., Hartig T., Hagerhall C.M., Fry G. (2009). Components of small urban parks that predict the possibility for restoration. Urban For. Urban Green..

[23] Pazhouhanfar M., Kamal M. (2014). Effect of predictors of visual preference as characteristics of urban natural landscapes in increasing perceived restorative potential. Urban For. Urban Green..

[24] Abkar M., Kamal M., Maulan S., Mariapan M., Davoodi S.R. (2011). Relationship between the preference and perceived restorative potential of urban landscapes. Hort Technol..

[25] Ayala-Azcárraga C., Diaz D., Zambrano L. (2019). Characteristics of urban parks and their relation to user well-being. Landsc. Urban Plan..

[26] Han K.T. (2010). An exploration of relationships among the responses to natural scenes: Scenic beauty, preference, and restoration. Environ. Behav..

[27] Johansson M., Gyllin M., Witzell J., Küller M. (2014). Does biological quality matter? Direct and reflected appraisal of biodiversity in temperate deciduous broad-leaf forest. Urban For. Urban Green..

[28] Sonntag-Öström E., Nordin M., Järvholm L.S., Lundell Y., Brännström R., Dolling A. (2011). Can the boreal forest be used for rehabilitation and recovery from stress-related exhaustion? A pilot study. Scand. J. For. Res..

[29] Sandifer P.A., Sutton-Grier A.E., Ward B.P. (2015). Exploring connections among nature, biodiversity, ecosystem services, and human health and well-being: Opportunities to enhance health and biodiversity conservation. Ecosyst. Serv..

[30] Hassan A., Chen Q.B., Jiang T. (2018). Physiological and psychological effects of gardening activity in older adults. Jpn. Geriatr. Soci..

[31] Tyrväinen L., Ojala A., Korpela K., Lanki T., Tsunetsugu Y., Kagawa T. (2014). The influence of urban green environments on stress relief measures: A field experiment. J. Environ. Psychol..

[32] Hankins T.C., Wilson G.F. (1998). A comparison of heart rate, eye activity, EEG and subjective measures of pilot mental workload during flight. Aviat. Space Environ. Med..

[33] Jensen O., Tesche C.D. (2002). Frontal theta activity in human increases with memory load in a working memory task. Eur. J. Neurosci..

[34] Kropotov J.D. (2009). Quantitative EEG, Event-Related Potentials and Neurotherapy.

[35] Ulrich R.S. (1981). Natural versus urban scenes: Some psychophysiological effects. Environ. Behav..

[36] Chang C.Y., Hammitt W.E., Chen P.K., Machnik L., Su W.C. (2008). Psychophysiological responses and restorative values of natural environments in Taiwan. Landsc. Urban Plan..

[37] Freeman D., Haselton P., Freeman J., Spanlang B., Kishore S., Albery E., Denne M., Brown P., Slater M., Nickless A. (2018). Automated psychological therapy using immersive virtual reality for treatment of fear of heights: A single-blind, parallel-group, randomised controlled trial. Lancet Psychiatry.

[38] Yu C.P., Lee H.Y., Luo X.Y. (2018). The effect of virtual reality forest and urban environments on physiological and psychological responses. Urban For. Urban Green..

[39] Birenboim A., Dijst M., Ettema D., Kruijf J.D., Leeuw G.D. (2019). The utilization of immersive virtual environments for the investigation of environmental preferences. Landsc. Urban Plan..

[40] Grassinia S., Revonsuoa A., Castellottia S., Petrizzoa I., Benedettia V., Koivistoa M. (2019). Processing of natural scenery is associated with lower attentional and cognitive load compared with urban ones. J. Environ. Psychol..

[41] Chang C.Y., Chen P.K. (2005). Human response to window views and indoor plants in the workplace. Hort Sci..

[42] Grove J.R., Prapavessis H. (1992). Preliminary evidence for the reliability and validity of an abbreviated Profile of Mood States. Int. J. Sport Psychol..

[43] Stroop J.R. (1935). Studies of interference in serial verbal reactions. J. Exp. Psychol..

[44] Wang H. (1997). Treatment of “don’t know” responses in contingent valuation surveys: A random valuation model. J. Environ. Econ. Manag..

[45] Russell J.A. (1980). A circumplex model of affect. J. Personal. Soc. Psychol..

[46] Chen H., Qiu L., Gao T. (2019). Application of the Eight Perceived Sensory Dimensions as a Tool for Urban Green Space Assessment and Planning in China. Urban For. Urban Green..

[47] Gale A., Coles M., Boyd E. (1971). Variation in visual input and the occipital EEG: II. Psychon Sci..

[48] Chiang Y.C., Li D.Y., Jane H.A. (2017). Wild or tended nature? The effects of landscape location and vegetation density on physiological and psychological responses. Landsc. Urban Plan..

[49] Barry J.R., Clarke A.R., Johnstone S.J., Magee C.A., Rushby J.A. (2007). EEG differences between eyes-closed and eyes-open resting conditions. Clin. Neurophysiol..

[50] Onishi K., Hagawara H. Effects of open versus closed eyes on physiological conditions during a working memory task. Proceedings of the 2017 IEEE 17th International Conference on Bioinformatics and Bioengineering.

[51] Beil K., Hanes D. (2013). The influence of urban natural and built environments on physiological and psychological measures of stress-A pilot study. Int. J. Environ. Res. Public Health.

[52] Berlyne D.E. (1960). Conflict, Arousal, and Curiosity.

[53] White M., Smith A., Humphryes K., Pahl S., Snelling D., Depledge M. (2010). Blue space: The importance of water for preference, affect, and restorativeness ratings of natural and built scenes. J. Environ. Psychol..

[54] Berman M., Jonides J., Kaplan S. (2008). The cognitive benefits for interacting with nature. Psychol. Sci..

[55] Muratet A., Pellegrini P., Dufour A.B., Arrif T., Chiron F. (2015). Perception and knowledge of plant diversity among urban park users. Landsc. Urban Plan..

[56] Petucco C., Skovsgaard J.P., Jensen F.S. (2013). Recreational preferences depending on thinning practice in young even-aged stands of pedunculate oak [Quercus robur L.]: Comparing the opinions of forest and landscape experts and the general population of Denmark. Scand. J. For. Res..

[57] Qiu L., Lindberg S., Nielsen A.B. (2013). Is biodiversity attractive?—On-site perception of recreational and biodiversity values in urban green space. Landsc. Urban Plan..

[58] Williams K.J., Cary J. (2002). Landscape preferences: Ecological quality and biodiversity protection. Environ. Behav..

[59] Nassauer J.I., Swaffield S. (2002). Messy ecosystems, orderly frames. Theory in Landscape Architecture: A Reader.

[60] Wilkie S., Clements H. (2018). Further exploration of environment preference and environment type congruence on restoration and perceived restoration potential. Landsc. Urban Plan..

[61] Vandierendonck A. (2017). A comparison of methods to combine speed and accuracy measures of performance: A rejoinder on the binning procedure. Behav. Res. Methods.

